# The Inverted Pyramid Approach: Early Combination Therapy With Voclosporin and Obinutuzumab in Active Lupus Nephritis

**DOI:** 10.7759/cureus.107549

**Published:** 2026-04-22

**Authors:** Homa Timlin, Abbal Koirala, Duruvu Geetha, Antoine Azar, Andrew Mener

**Affiliations:** 1 Rheumatology, Johns Hopkins University School of Medicine, Baltimore, USA; 2 Nephrology, Johns Hopkins Bayview Medical Center, Baltimore, USA; 3 Nephrology, Johns Hopkins University School of Medicine, Baltimore, USA; 4 Allergy and Immunology, Johns Hopkins University School of Medicine, Baltimore, USA; 5 Oncology, Maryland Oncology Hematology, Columbia, USA

**Keywords:** lupus nephritis, obinutuzumab, proteinuria, systemic lupus erythematosus, voclosporin

## Abstract

Achieving remission or low disease activity in systemic lupus erythematosus reduces long-term organ damage, disease flares, and corticosteroid exposure. Kidney involvement from lupus nephritis is often progressive and associated with adverse long-term outcomes. Early, effective immunosuppression during the active inflammatory phase of lupus nephritis is linked to improved renal outcomes. Although voclosporin and obinutuzumab have each demonstrated efficacy in lupus nephritis, real-world data on their combined use remain limited. Here, we report outcomes in three patients with active lupus nephritis who were treated with both Food and Drug Administration (FDA)-approved agents.

## Introduction

Lupus nephritis (LN) affects patients with systemic lupus erythematosus (SLE) across all age groups, with a U.S. prevalence of 30.9 per 100,000 [1]. Delayed diagnosis and treatment are associated with worse outcomes and increased risk of end-stage kidney disease (ESKD). Early reduction in proteinuria strongly predicts long-term renal preservation, reflecting the greater reversibility of injury during the initial inflammatory phase [2,3]. Recurrent LN flares drive cumulative nephron loss and progression to ESKD, which occurs in 10%-22% of patients [4,5].

These data support early use of immunosuppression in active LN, aiming to achieve effective disease control, reduce proteinuria, and preserve kidney function. Given the multifactorial pathogenesis of LN, targeting complementary inflammatory and podocyte injury pathways is a rational therapeutic strategy. Controlling active LN requires potent immunosuppressive drugs. Adding B-cell-targeting therapies or calcineurin inhibitors can result in increased frequencies of complete responses and a faster treatment response.

## Case presentation

Subject 1

An African American female patient was diagnosed with SLE at age 31, characterized by positive ANA, anti-Smith, anti-double-stranded DNA (anti-dsDNA), anti-β2 glycoprotein I (IgG), Coombs positivity, hypocomplementemia, LN (class IV, May 2018; class III, June 2025), and arthritis. Supporting features included sicca symptoms, elevated ESR, Raynaud’s phenomenon, and possible lymphadenopathy.

At initial diagnosis of class IV LN in 2018, she received intravenous (IV) solumedrol 1 g daily for three consecutive days, followed by oral prednisone 60 mg with taper, in combination with mycophenolate mofetil (MMF) and hydroxychloroquine (HCQ). Proteinuria resolved from 4 to <0.2 g within eight weeks, and steroids were successfully tapered off over the next 19 months.

Over the subsequent six years, she experienced intermittent inflammatory arthralgia responsive to short courses of steroids. She received azathioprine (Imuran) for two years but was unable to tolerate belimumab due to pruritus. Laboratory monitoring during this period revealed intermittent positive anti-dsDNA, normal C3 and C4, persistently normal urine protein-to-creatinine ratio (UPCR < 0.2), and estimated glomerular filtration rate (eGFR) > 60 mL/min/1.73 m².

In 2025, she experienced a flare of arthritis with increasing proteinuria, peaking at 2.08 g. Laboratory evaluation showed positive anti-dsDNA, low C3, borderline low serum albumin at 3.4 g/dL (normal value 3.5-5 g/dL), and eGFR > 60 mL/min/1.73 m². A repeat kidney biopsy confirmed class III LN. She was treated with IV solumedrol 500 mg daily for three consecutive days, followed by prednisone 20 mg daily, voclosporin (Lupkynis) 23.7 mg twice daily, and reinitiation of MMF 1,500 mg twice daily. The patient self-reduced prednisone by 5 mg, and due to gastrointestinal intolerance, MMF was switched to mycophenolic acid (Myfortic) 1,080 mg twice daily.

**Figure 1:**
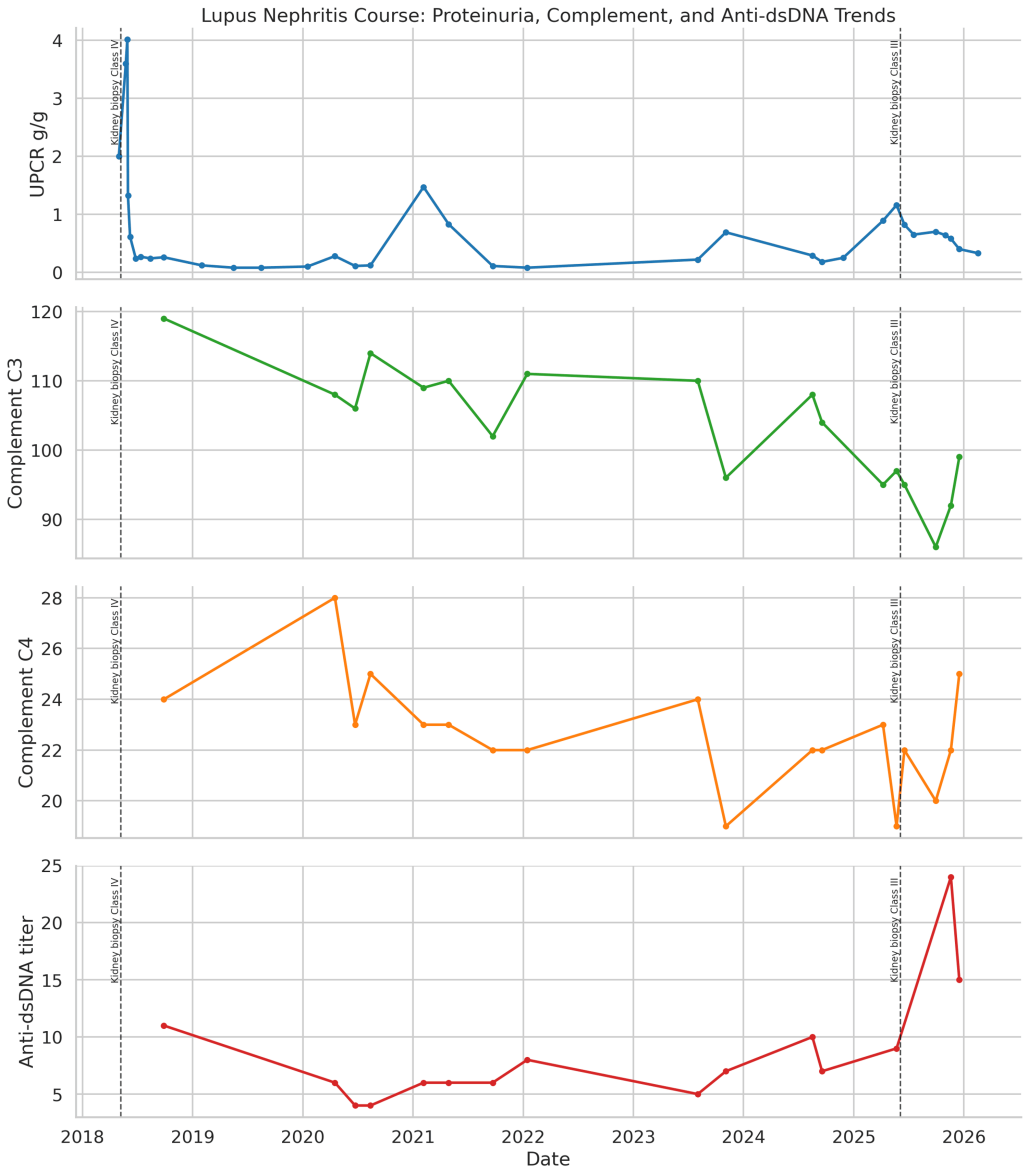
Longitudinal laboratory trends during the course of lupus nephritis, including urine protein-creatinine ratio (UPCR), complement C3, complement C4, and anti-double-stranded DNA (anti-dsDNA) titers for Case 1. Vertical dashed lines indicate the timing of kidney biopsies in May 2018 and June 2025, demonstrating class IV and class III lupus nephritis, respectively. The figure shows severe proteinuria at initial presentation, prolonged improvement after induction therapy, recurrent proteinuria in 2025, and subsequent improvement following treatment intensification with glucocorticoids, voclosporin, mycophenolate-based therapy, and obinutuzumab.

**Figure 2:**
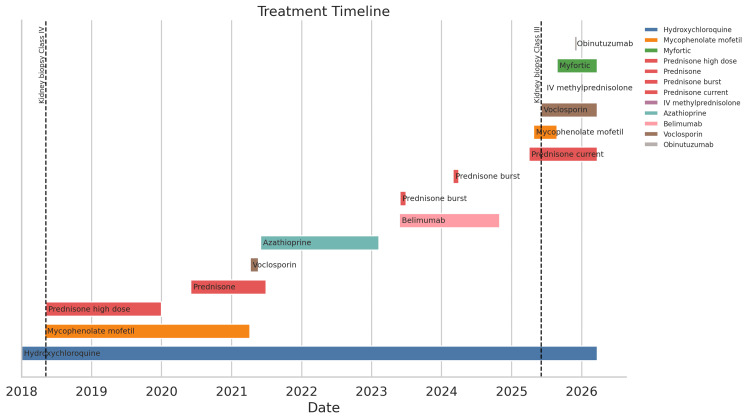
Timeline of immunomodulatory therapy across the patient’s lupus nephritis course for Case 1 Bars represent treatment exposure to hydroxychloroquine, mycophenolate mofetil, mycophenolic acid, glucocorticoids, azathioprine, belimumab, voclosporin, intravenous (IV) methylprednisolone, and obinutuzumab. Vertical dashed lines indicate the timing of kidney biopsies in May 2018 and June 2025. The timeline shows initial induction therapy for class IV lupus nephritis, a prolonged period of relative renal stability, and, in 2025, the escalation of immunosuppression for recurrent class III lupus nephritis.

**Figure 3:**
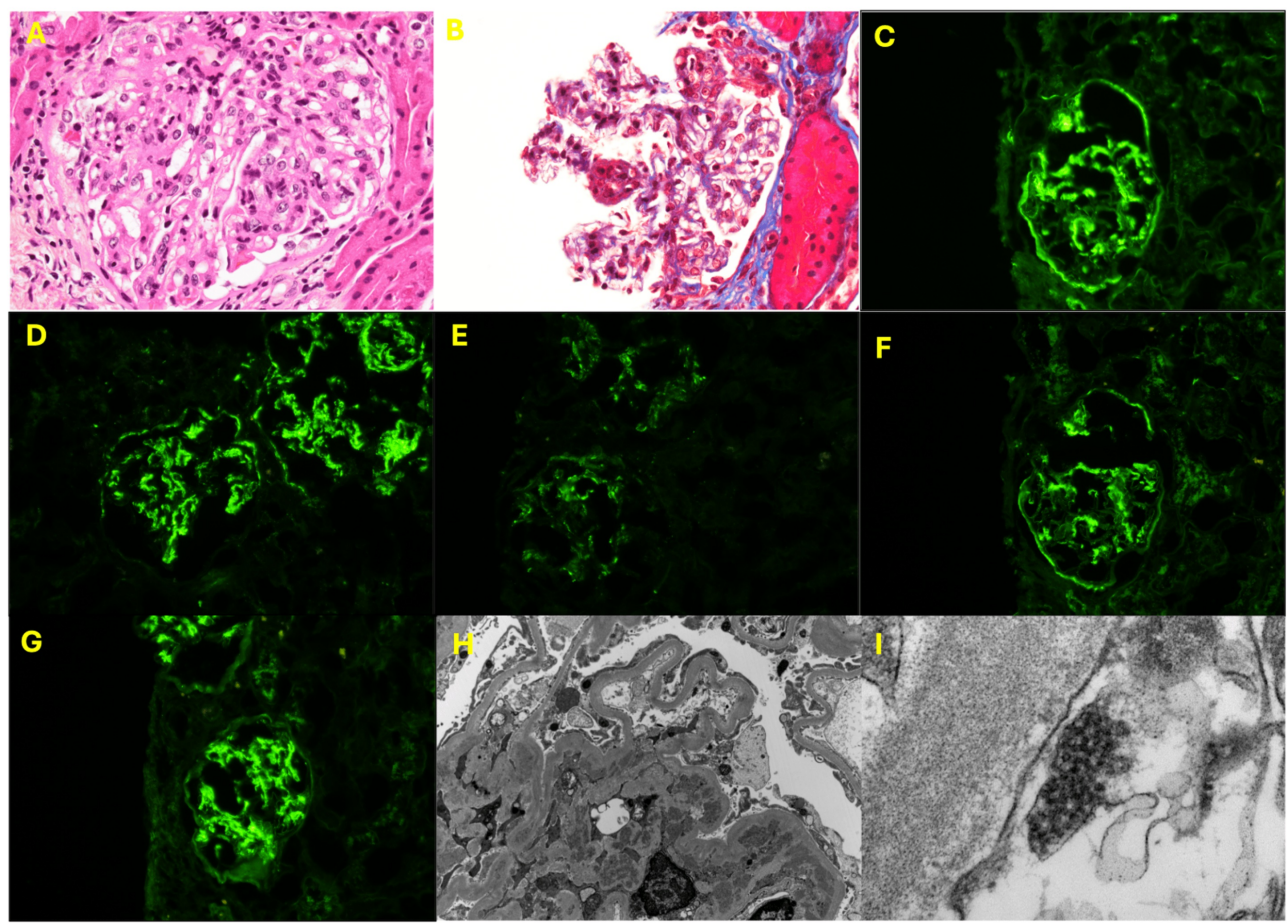
Kidney biopsy findings of Case 1: (A, B) light microscopy, (C-G) immunofluorescence (IF), and (H, I) electron microscopy (EM) (A) Mesangial hypercellularity; (B) endocapillary hypercellularity; (C) IF for IgA; (D) IF for C3; (E) IF for C1q; (F) IF for kappa; (G) IF for lambda; (H) mesangial immune deposits on EM; (I) tubuloreticular inclusions

Following treatment intensification with obinutuzumab (1 g two weeks apart), in combination with voclosporin (held mycophenolic acid during her infusions), her arthritis resolved, and proteinuria decreased from 2 to 0.4 g within less than two weeks after her last infusion. At her most recent visit, two months after the last obinutuzumab dose, she remained clinically stable on HCQ 400 mg daily, prednisone 5 mg daily, voclosporin 23.7 mg twice daily, and mycophenolic acid 1,080 mg twice daily (see Figures 1-3).

Subject 2

A Caucasian female patient developed arthralgia at age 16 and was diagnosed with SLE after the birth of her first child. Her disease was characterized by positive ANA, hypocomplementemia, LN (class V in 2015; class IV/V in August 2024), alopecia, arthritis, biopsy-proven lupus rash, and mucosal ulcers. Supporting features included Raynaud’s phenomenon.

Following her initial LN diagnosis in 2015, she was treated with steroids, MMF (1,500 mg twice daily), and HCQ (400 mg daily). In early 2024, voclosporin (23.7 mg twice daily) was briefly attempted but discontinued due to rash. Repeat biopsy in August 2024 confirmed class IV/V LN, and proteinuria persisted despite monthly belimumab infusions.

At second opinion, she had active disease on HCQ 200 mg BID, MMF (1,500 mg twice daily), prednisone 10-20 mg, monthly belimumab, and daily candesartan. Labs showed UPCR 1.8 g, normal serum albumin, eGFR > 60 mL/min/1.73 m², negative dsDNA, and normal complements. She tolerated voclosporin upon slow re-titration. MMF was switched to mycophenolic acid 1,080 mg twice daily due to gastrointestinal intolerance.

**Figure 4:**
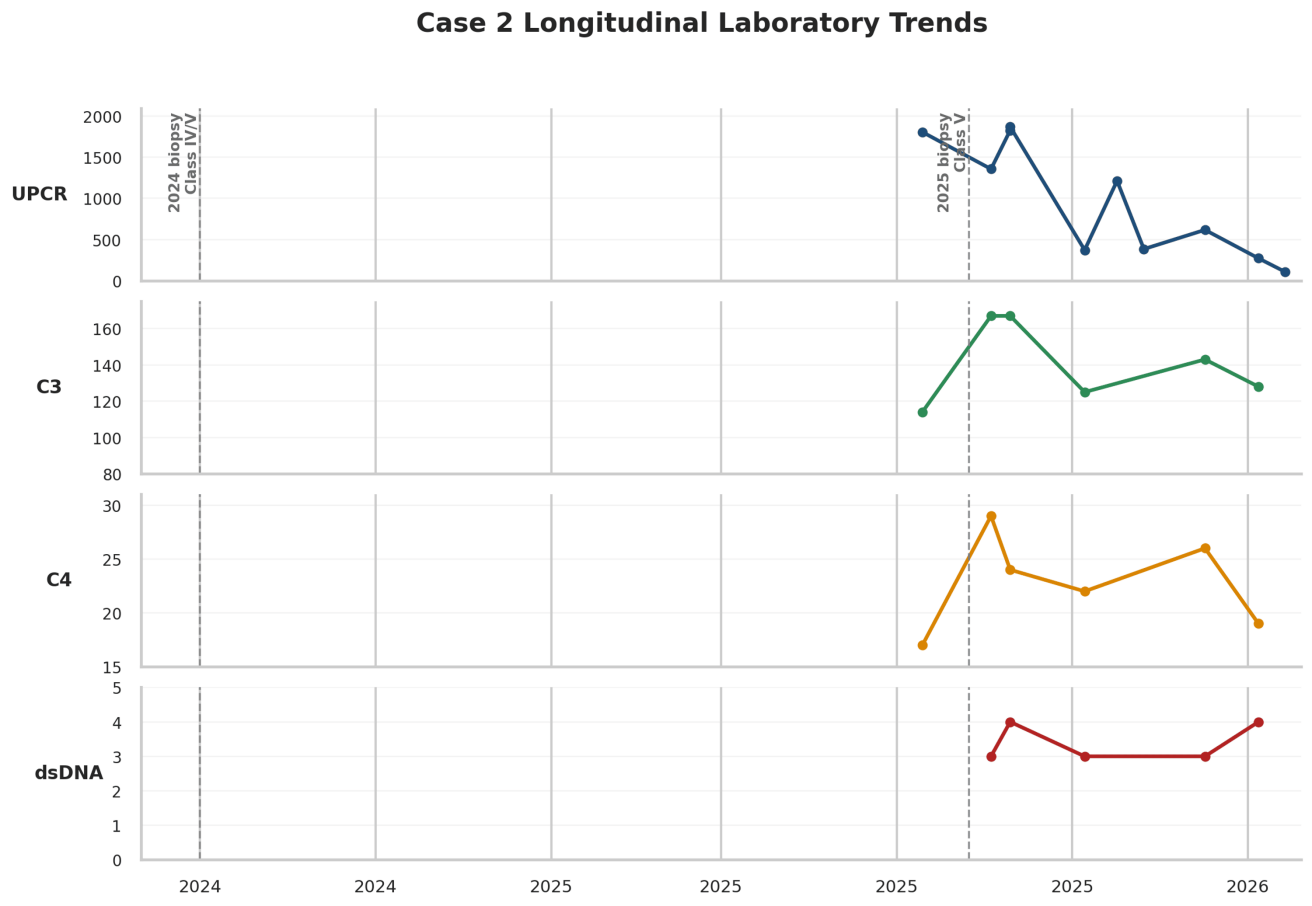
Longitudinal laboratory trends during the course of lupus nephritis, including urine protein-creatinine ratio (UPCR), complement C3, complement C4, and anti-double-stranded DNA (anti-dsDNA) titers for Case 2 Vertical dashed lines indicate the timing of kidney biopsies in 2015, August 2024, and September 2025, demonstrating class V, class IV/V, and class V lupus nephritis, respectively. The figure shows persistent proteinuria in 2025, with UPCR 1.804 g/g in August 2025 and a peak recorded value of 1.867 g/g on September 29, 2025, followed by improvement after reintroduction of voclosporin, pulse glucocorticoids, and transition to obinutuzumab-based therapy, reaching 0.107 g/g on February 20, 2026. Throughout the documented interval, C3 and C4 remained within the normal range, and anti-dsDNA titers stayed low-level positive.

**Figure 5:**
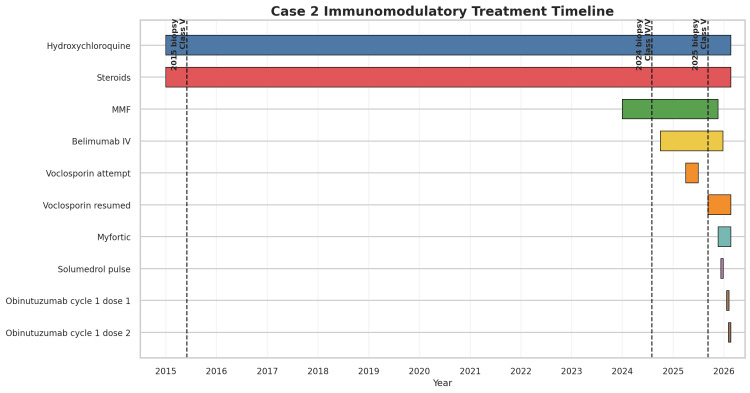
Timeline of immunomodulatory therapy across the patient’s lupus nephritis course for Case 2 Bars represent treatment exposure to hydroxychloroquine, mycophenolate mofetil (MMF), mycophenolic acid, glucocorticoids, voclosporin, belimumab, intravenous (IV) methylprednisolone, and obinutuzumab. Vertical dashed lines indicate the timing of kidney biopsies in 2015, August 2024, and September 2025. The timeline demonstrates long-term background therapy after initial class V lupus nephritis, escalation for class IV/V disease in 2024, and subsequent transition from belimumab to obinutuzumab for persistent active class V lupus nephritis in 2025-2026.

**Figure 6:**
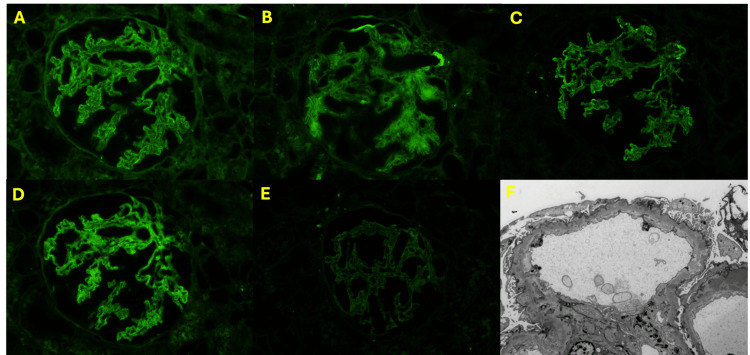
Kidney biopsy findings: (A-E) immunofluorescence (IF) and (F) electron microscopy (EM) (A) IF for IgG; (B) IF for C3; (C) IF for C1q; (D) IF for kappa; (E) IF for lambda; (F) EM with subepithelial and intramembranous deposits with resorption.

A repeat biopsy confirmed class V LN. She received solumedrol 500 mg IV × 2 days, and belimumab was switched to obinutuzumab (1 g two weeks apart, held mycophenolic acid during her infusions). Three weeks after her last infusion, she had significant improvement in morning stiffness, residual synovitis in one joint, and improved lupus rash. Labs revealed UPCR < 0.2 g, eGFR > 60, negative dsDNA, and normal complements. She was continued on prednisone 5 mg daily, HCQ 400 mg, mycophenolic acid 1,080 mg twice daily, and voclosporin 23.7 mg twice daily. Candesartan dose was increased, and nifedipine was added for improved blood pressure and Raynaud’s control (see Figures 4-6).

Subject 3

An African American female patient was diagnosed with SLE in 2025 at age 32, characterized by positive ANA, anti-Smith, discoid rash, proteinuria (peak UPCR 2.9 g on July 2025), LN (class III on July 2025 and December 25), leukopenia, nasal ulcers, alopecia, and arthritis. Supporting features included positive chromatin antibodies.

Initial therapy included HCQ 400 mg, IV solumedrol (500 mg for three consecutive days), prednisone (starting at 40 mg), MMF 3 g, and rituximab (one dose of 1,000 mg). Belimumab was not used due to insurance denial. Proteinuria improved with voclosporin 23.7 mg BID (UPCR decreased from 2.2 to 0.8 g), but the patient later self-discontinued MMF, possibly due to gastrointestinal intolerance. Mycophenolic acid could not be initiated due to high cost. Repeat kidney biopsy confirmed class III LN.

**Figure 7:**
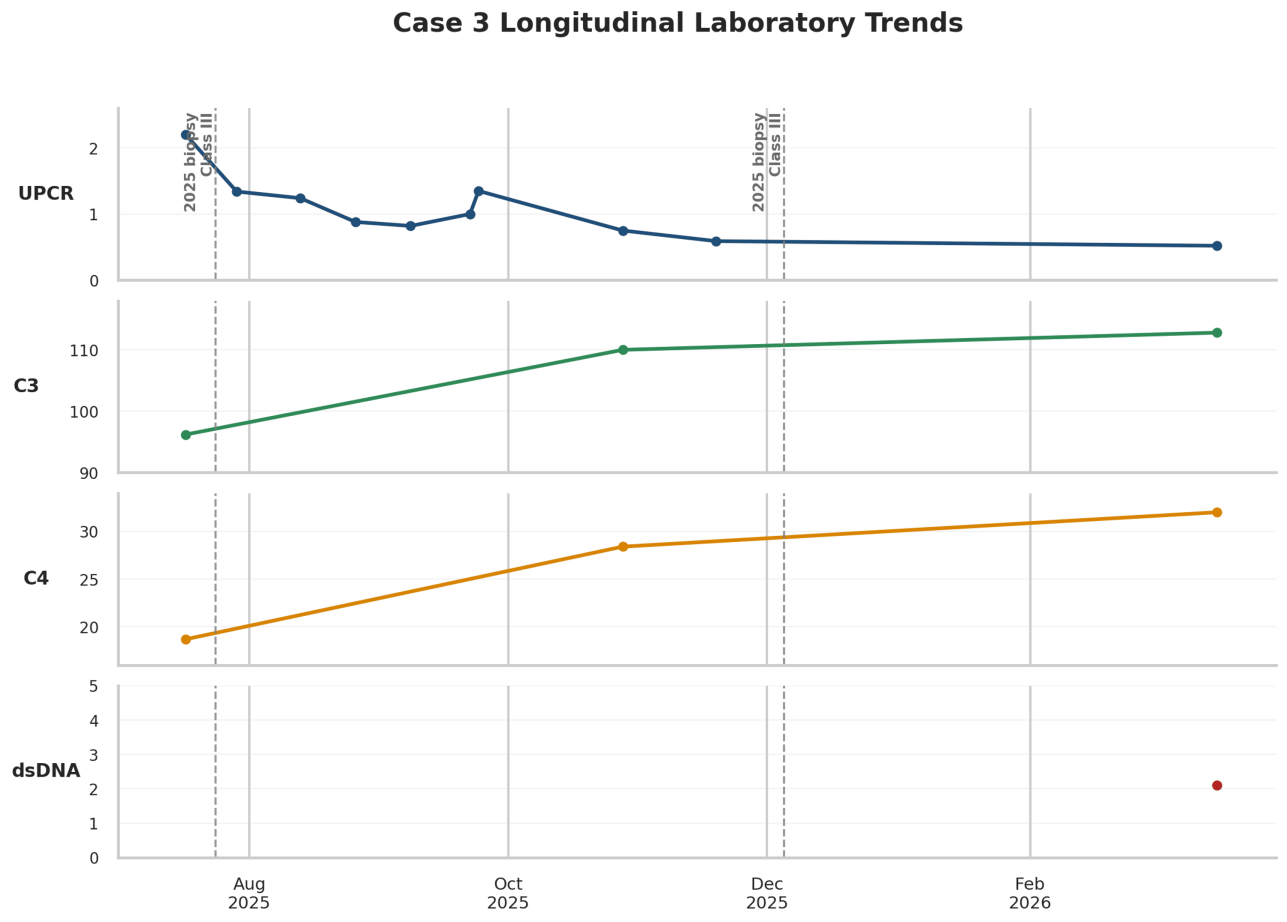
Longitudinal laboratory trends during the course of lupus nephritis, including urine protein-creatinine ratio (UPCR), complement C3, complement C4, and anti-double-stranded DNA (anti-dsDNA) titers for Case 3 Serial laboratory measurements in Case 3 showing UPCR, complement C3, complement C4, and anti-dsDNA over time from July 2025 through March 2026. Vertical dashed lines indicate the timing of kidney biopsies, both of which demonstrate class III lupus nephritis. UPCR decreased over follow-up, while complement levels normalized, and dsDNA was negative at the latest assessment, consistent with improving serologic and renal disease activity.

**Figure 8:**
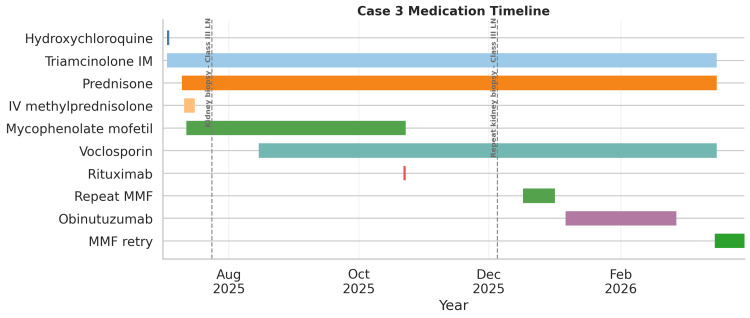
Gantt-style timeline of immunomodulatory and lupus nephritis-directed therapies in Case 3 Gantt-style timeline of immunomodulatory and lupus nephritis-directed therapies in Case 3, including hydroxychloroquine, glucocorticoids, mycophenolate mofetil (MMF), voclosporin, rituximab, and obinutuzumab. Vertical dashed lines mark the timing of kidney biopsies, both of which are labeled class III lupus nephritis. This figure illustrates the sequence, overlap, and duration of therapies relative to biopsy timing and clinical course. IV: intravenous; IM: intramuscular

**Figure 9:**
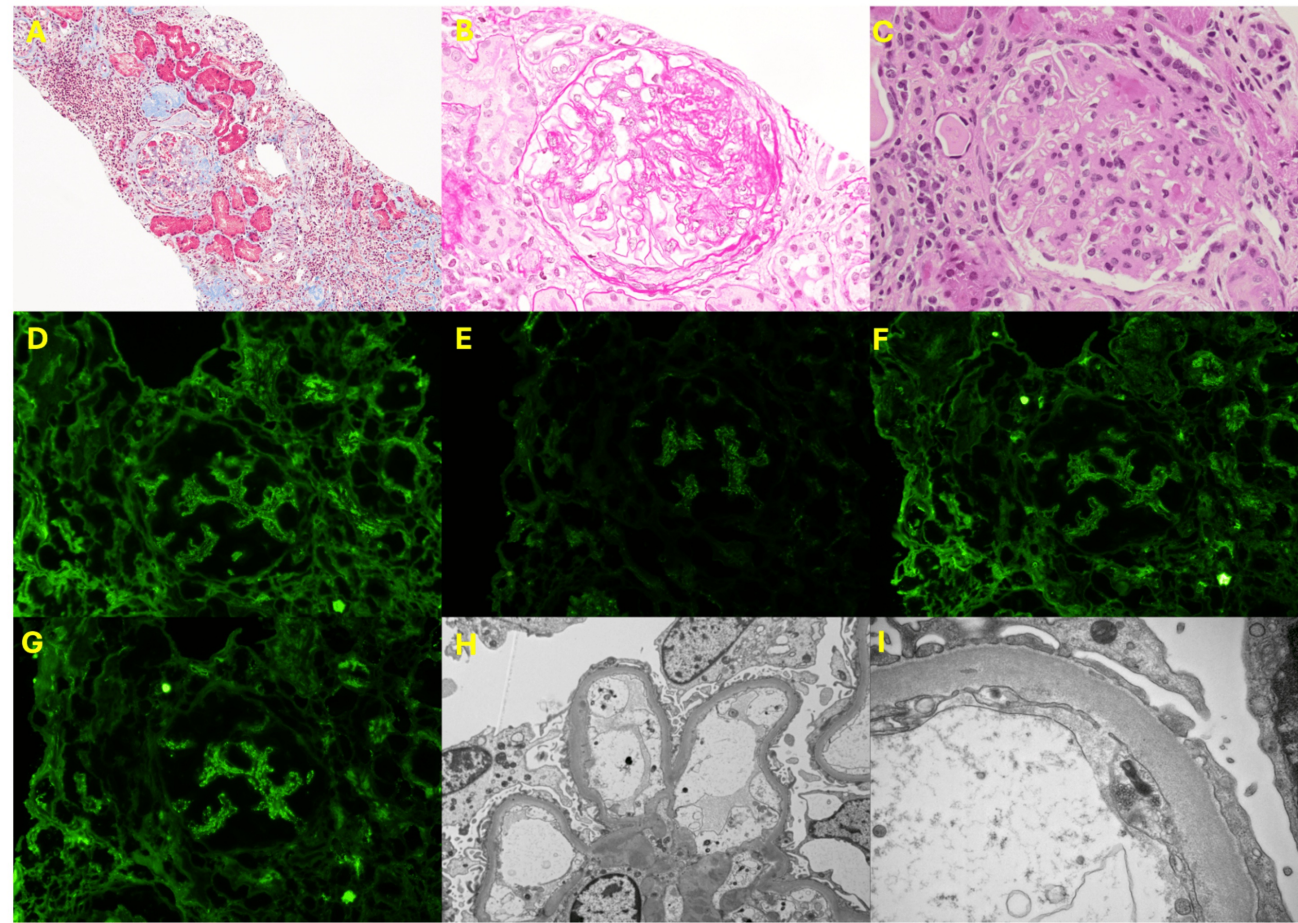
Kidney biopsy findings of Case 1: (A-C) light microscopy, (D-G) immunofluorescence (IF), and (H, I) electron microscopy (EM) (A) Tubulointerstitial inflammation, scarring, global and segmental glomerulosclerosis; (B) focal segmental glomerulosclerosis (FSGS); (C) mesangial hypercellularity; (D) IF for IgG; (E) IF for C3; (F) IF for kappa; (G) IF for lambda; (H) mesangial deposits on EM; (I) tubuloreticular inclusions (TRI) in endothelial cells.

Due to persistent polyarthritis and proteinuria, obinutuzumab was initiated (1 g two weeks apart). Twelve weeks after her last infusion, she had improvement in arthritis, with involvement of only one joint and minimal arthralgia. Labs showed UPCR 0.52 g, eGFR > 60 mL/min/1.73 m², normal complements, and negative dsDNA. Current therapy included HCQ 200 mg BID, prednisone 5 mg daily, voclosporin 23.7 mg BID, and resumed MMF titrated toward 1,000 mg BID (see Figures 7-9; Tables 1-3).

**Table 1:** Subject 1 Data presented above show laboratory values before and after drug administration. Dates correspond to when medications were administered or laboratory tests were obtained. The UPCR trended downward over follow-up, accompanied by improvement in leukopenia and neutropenia. UPCR: urine protein-to-creatinine ratio; WBC: white blood cell count; ANC: absolute neutrophil count; N/A: data not available

Patient values					
Parameters	Within 2 weeks before voclosporin	Within 6 weeks after voclosporin	Within 1 week before obinutuzumab	Within 8 weeks after obinutuzumab	Reference range
UPCR	0.80 g	0.11 g	1.1 g	0.35 g	<0.2 g
Immunoglobulin IgM	-	-	N/A	127	35-242 mg/dL
Immunoglobulin IgG	-	-	N/A	1,121	610-1,616 mg/dL
Immunoglobulin IgA	-	-	N/A	236	61-348 mg/dL
WBC	-	-	3.3	4.07	4.5-11 K/cu mm
ANC	-	-	1.3	1.74	1.5-7.8 K/cu mm

**Table 2:** Subject 2 Data presented above show laboratory values before and after drug administration. Dates correspond to when medications were administered or laboratory tests were obtained. The UPCR trended downward over follow-up without development of leukopenia or neutropenia. UPCR: urine protein-to-creatinine ratio; WBC: white blood cell count; ANC: absolute neutrophil count; N/A: data not available

Patient values					
Parameters	Within 3 weeks before voclosporin	Within 8 weeks after voclosporin	Within 2 weeks before obinutuzumab	Within 6 weeks after obinutuzumab	Reference range
UPCR	1.80 g	0.37 g	0.61 g	0.23 g	<0.2 g
Immunoglobulin IgM	-	-	33	N/A	26-217 mg/dL
Immunoglobulin IgG	-	-	1,161	N/A	586-1,602 mg/dL
Immunoglobulin IgA	-	-	133	N/A	87-352 mg/dL
WBC	-	-	4.9	5.8	3.4-10.8 x 10^3^/µL
ANC	-	-	3.1	4	1.4-7 x 10^3^/µL

**Table 3:** Subject 3 Data presented above show laboratory values before and after drug administration. Dates correspond to when medications were administered or laboratory tests were obtained. The UPCR trended downward over follow-up, with only mild leukopenia and neutropenia observed. UPCR: urine protein-to-creatinine ratio; WBC: white blood cell count, ANC: absolute neutrophil count

Patient values					
Parameters	Within 2 weeks before voclosporin	Within 3 weeks after voclosporin	Within 4 weeks before obinutuzumab	Within 12 weeks after obinutuzumab	Reference range
UPCR	1.24 g	1 g	0.59 g	0.52 g	<0.2 g
Immunoglobulin IgM	-	-	83	92	35-242 mg/dL
Immunoglobulin IgG	-	-	1,190	1,372	610-1,616 mg/dL
Immunoglobulin IgA	-	-	246	249	61-348 mg/dL
WBC	-	-	3.65	2.6	4.5-11 K/cu mm
ANC	-	-	2.19	1.43	1.5-7.8 K/cu mm

## Discussion

Early response to immunosuppressive therapy predicts favorable long-term renal outcomes in LN [4,5]. The 2024 American College of Rheumatology guidelines recommend triple therapy for active LN, typically comprising glucocorticoids, MMF, and either belimumab or a calcineurin inhibitor [6]. In 2025, the U.S. Food and Drug Administration approved obinutuzumab for adults with active LN receiving standard therapy. Obinutuzumab, a type II glycoengineered anti-CD20 monoclonal antibody, depletes B-cells via enhanced antibody-dependent cellular cytotoxicity, direct cell death, and phagocytosis, reducing autoantibody production and immune complex formation [7]. Voclosporin, a novel oral calcineurin inhibitor, acts by suppressing T-cell activation through IL-2 inhibition and stabilizing the podocyte cytoskeleton, resulting in rapid proteinuria reduction. Together, these agents target complementary immune pathways, combining rapid hemodynamic and immunologic effects with sustained modulation of B-cell-driven autoimmunity [8]. The ALLEGORY study further demonstrated that obinutuzumab significantly reduces disease activity in adults with SLE [9].

Safety and tolerability remain critical considerations, with a key treatment goal being the minimization of long-term corticosteroid exposure without triggering LN flares. In our series of three patients with active LN, combination therapy was well tolerated, produced rapid improvements in disease activity and proteinuria, and allowed successful glucocorticoid tapering without serious infections thus far. Limitations include the retrospective design, small sample size, and short follow-up, which preclude assessment of long-term renal outcomes and safety. Larger clinical trials are needed to confirm the safety and efficacy of this strategy.

## Conclusions

In this case series, combined voclosporin and obinutuzumab during the active phase of LN was associated with improvement in proteinuria and disease activity over follow-up. The combination was well tolerated in these cases and supported glucocorticoid tapering without serious infectious complications during the available observation period. These findings suggest a potential role for early combination therapy in enhancing disease control. Further studies are needed to assess the efficacy and safety of this combination therapy.
